# Antioxidant and Protective Effects of *Artemisia campestris* Essential Oil Against Chlorpyrifos-Induced Kidney and Liver Injuries in Rats

**DOI:** 10.3389/fphys.2021.618582

**Published:** 2021-02-24

**Authors:** Mongi Saoudi, Riadh Badraoui, Fatma Rahmouni, Kamel Jamoussi, Abdelfattah El Feki

**Affiliations:** ^1^Animal Ecophysiology Laboratory, Sciences Faculty of Sfax, University of Sfax, Sfax, Tunisia; ^2^Department of Biology, University of Hai’l, Ha’il, Saudi Arabia; ^3^Laboratory of Histology - Cytology, Medicine Faculty of Tunis, University of Tunis El Manar, Tunis, Tunisia; ^4^Laboratory of Histo-Embryology and Cytogenetics, Medicine Faculty of Sfax University, Sfax, Tunisia; ^5^Biochemistry Laboratory, University Hospital Complex (CHU) Hedi Chaker of Sfax, Sfax, Tunisia

**Keywords:** chlorpyrifos toxicity, kidney injury, liver injury, oxidative stress, *Artemisia campestris* essential oil

## Abstract

This study is aimed to elucidate the possible antioxidant and protective effects of *Artemisia campestris* essential oil (ACEO) against the deleterious effects of chlorpyrifos (CPF) in rats. The *in vivo* study revealed increases in aspartate aminotransferase (AST), alanine aminotransferase (ALT), lactate dehydrogenase (LDH), and alkaline phosphatase (ALP) activities and the serum contents of creatinine, urea, uric acid, cholesterol, triglycerides, low density lipoproteins (LDL), and glucose in rats treated with CPF as compared to controls. Meanwhile, hepatic and renal activities of superoxide dismutase (SOD), catalase (CAT), and glutathione peroxidase (GPx) in liver and kidney decreased and the content of malondialdehyde (MDA) increased. Some histopathologic features were noticed in liver and kidney of the CPF group. Interestingly, ACEO alleviated the biochemical disruptions and reduced these hepato-renal morphologic changes.

## Introduction

Pesticides are commonly used to control insects, fungal diseases, and weeds to protect the world’s food supply. They are particularly used in agriculture to protect crops, but also in many other professional activities (maintenance of green spaces, wood treatment, disinfection of premises, veterinary uses.) as well as for domestic uses (de Bon et al., 2014). Indeed, humans are differently exposed to pesticides; either directly in agricultural occupations or indirectly through the consumption of contaminated food (Badraoui et al., 2007; Negatu et al., 2016; Mzid et al., 2017). The appearance of these molecules (pesticides), generally more effective against pests than the inorganic molecules that preceded them (copper sulfate, lime.). Hence, these products have made interesting progress in the quality and quantity of crops and in the fight against disease vectors (Donkor et al., 2016). However, their wide diffusion and toxic potential raised several questions about their risks to human health. Pesticides may enter the body when the product is mixed, applied or cleaned. Also, pesticides can enter the body via the three major routes of exposure: inhalation, ingestion, and skin contact through the lungs, the mouth, and the dermal contact, respectively. Pesticides pose a real public health problem (Badraoui et al., 2007, 2020; Mzid et al., 2017; Rahmouni et al., 2019). Indeed, the effects of low amounts of pesticides, in mixture, for long periods pose many health problems. Recent studies showed that people exposed to pesticides are more likely to develop cancer, congenital malformations, infertility problems, neurological problems or weakened immune systems than others (Sears et al., 2006; Badraoui et al., 2007, 2012; Mzid et al., 2017). Chlorpyrifos (CPF) is one of the most widely used organophosphorus (OP) in the world for both agriculture and indoor applications (Sharma and Chadha, 2016). The active substance in CPF induces a disruption of nervous system function by inhibition of acetylcholinesterase (AChE), an enzyme that degrades the acetylcholine (Ach) neurotransmitter in both neuromuscular junctions and cholinergic synapses (Abolaji et al., 2017b). The AChE enzyme is common to many animals which confers on OPs a relative selectivity. For this reason, the activity of CPF is not limited to insects. For example, OPs are very dangerous to ecosystems (invertebrates and fish), even at very low doses. The neurotoxic effects of CPF are related to the cholinergic function, which in turn induces hyperactivity in the cholinergic synapses (Albuquerquea et al., 2017). These neurotoxic effects are not related to CPF itself but to its main metabolite; the chorpyifos-oxon. The latter is well-known to bind to the enzyme acetylcholinesterase and blocking/disabling its activity within the nervous synapses (Abolaji et al., 2017a,b). Also, CPF produces many other damaging effects, such as embryo- and geno- toxicity, immunological disruptions, hepatic failure, teratogenicity and neurobehavioral changes (Burke, 2016). Recently, many studies demonstrated that oxygen free radicals play an important role in the pesticides’ toxicity (Asghari et al., 2017; Mzid et al., 2017; Rahmouni et al., 2019; Badraoui et al., 2020). It has been reported in an experimental study, that CPF produced oxidative damage associating accumulation of lipid peroxidation products in several organs (Tanvir et al., 2016). CPF may differentially change the activity of the cell antioxidants such assuperoxide dismutase (SOD), glutathione peroxidase (GPx), and catalase (CAT). Thus, could result in of oxidative stress damage in various organ systems. Recent study by Narra et al. (2017) reported that CPF significantly decreased the activities of these antioxidant enzymes (SOD, CAT, and GPx). Such effects of CPF might associate the perturbation of the normal cellular development and/or differentiation (Dominah et al., 2017). Repeated exposure to CPF caused significant hepato-renal toxicity. In fact, several disruptions in profile of both liver and kidney markers have been reported. Together with this, essential trace elements and antioxidant enzymes were found to be adversely affected in CPF intoxicated animals (Abolaji et al., 2017a).

Several dietary antioxidant compounds have been reported to have health benefits. The consumption of these products decreased not only oxidative damage but also pro-inflammatory biomarkers. It is believed that antioxidants, such as vitamins, can neutralize the formation of oxygen free radicals or overcome their chemico-biological results, particularly their excessive formation which is commonly associated with deleterious effects. Likewise, non-enzymatic antioxidants including vitamins E and C, can inhibit oxidative stress development via their important scavenging potential. It has also been shown that vitamin C can react with the radicals of vitamin E to regenerate vitamin E (Badraoui et al., 2009; Rahmouni et al., 2019). Another sources of antioxidants, the herbalism which is a promising and ethno-pharmacological practice (Akacha et al., 2020). It refers to the use of a particular plant or one of its parts, its extracts and/or oil in the treatment of some pathologies or discomforts. *Artemisia campestris* (family: Asteraceae), also called “Tegoufet” in Tunisia, has been long used as traditional medicine. It has been widely used as an antivenin, anti-inflammatory, anti-microbial properties (Akrout et al., 2011), and antispasmodic, antiseptic and for the restoration of declining mental function and inflammation of the liver (Dib et al., 2017). The *A. campestris* essential oil (ACEO) has been extracted and its phytochemical composition was previously investigated. Furthermore, it has been reported that ACEO is a rich source of antioxidants and bioactive compounds (Saoudi et al., 2017a).

The role of this promising ACEO against CPF-induced changes in hepatic and renal systems has not been investigated so far. Hence, the current study was undertaken to assess the potential antioxidant and protective effects of ACEO. Its effects on CPF-induced liver and kidney toxicity were also explored in adult male rats.

## Materials and Methods

### Chemicals

The chlorpyrifos (diethyl 3,5,6,-tricholoro-2-pyridyl phosphorothionate, formulation EC48%) was bought from the Sfax company of materials, seeds and agricultural products. The other chemical products were of analytical grades and purchased from Sigma^®^ Chemicals Company.

### Plant Material and Extraction of Essential Oil

*A. campestris* aerial parts (stem and leaves) were collected from Kasserine (Central region of Tunisia). Small pieces of the plant were subjected to 2 h hydro distillation using a Clevenger-type apparatus. The oil phase was extracted and run three successive times using diethyl ether, then dried using a vaccum. The extracted essential oil was then purified and stored until further use.

### Animals

#### Rats Farming

Male *Wistar* rats of about 210 g were purchased from the Central Pharmacy of Tunisia (SIPHAT, Tunisia). The rats were given a standard diet provided by SICO company (Sfax, Tunisia) and drinking water *ad libitum*. The animals were housed under well-controlled conditions of temperature (22 ± 3°C), 12 h light/dark cycles and humidity (40%). All animal experiments were approved and carried out according to the guidelines of our institution Ethical Committee for care and use of laboratory animals.

#### Experimental Protocols

**FIGURE 1:**
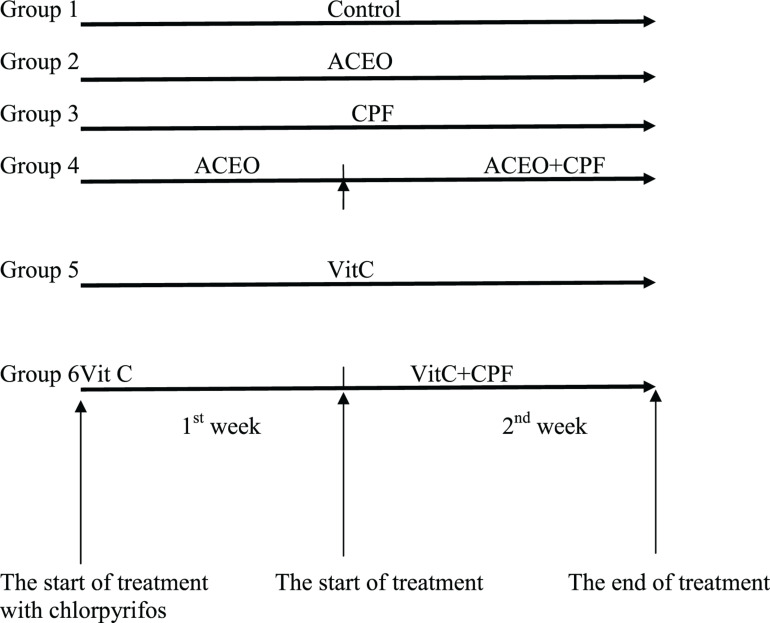
The experimental scheme.

After acclimatization, the animals were randomly divided into six groups (Figure 1) of six rats each. All the treatments were given in the morning between 9 and 10 am.

Group I: Control rats (C group) received drinkable water, standard diet, water *ad libitum* and corn oil was intra-peritoneally injected (IP) for 2 weeks.

Group II: The rats were treated by 200 mg/kg b.w. ACEO (ACEO group) by IP during 2 weeks. Both dose and period were selected according to previous published study (Radulović et al., 2013).

Group III: Chlorpyrifos (CPF group) was given per gavage for 2 weeks at a dose of 63 mg/kg b.w. (Bebe and Panemangalore, 2003).

Group IV: Rats were pre-treated by ACEO during 2 weeks and then CPF was applied together with ACEO during the second week (ACEO + CPF group).

Group V: Vitamin C at the dose of 200 mg/kg b.w. (Petrova et al., 1992) was IP-injected to rats during 2 weeks (Vit C group).

Group VI: Before CPF exposure, rats received an IP pre-treatment with vitamin C (200 mg/kg b.w.) during 2 weeks and then CPF was given along with vitamin C for the second week. ACEO (dissolved in corn oil) was administered to the concerned groups. The food intake and the body weight variations were followed.

### Tissues Collection and Preparation

After 2 weeks of treatments, the rats were sacrificed by decapitation under general anesthesia. Serum samples were collected from the blood of each rat by centrifuging at 2,700 g for 15 min. Both kidneys and livers were also collected. For each rat, kidney and liver specimens were homogenized with a Potter (glass-teflon) in the presence of 10 mM Tris-HCl, pH 7.4. The homogenates were centrifuged at 4°C at 10,000 g for 15 min using Ultra Turrax (T25, Germany). Aliquots of serum and tissue supernatants were kept at −30°C until analyzed.

### Serum Markers

Aspartate and alanine aminotransferases (AST and ALT, respectively), alkaline phosphatase (ALP), lactate dehydrogenase (LDH), creatinine, urea, glucose, uric acid, cholesterol, triglycerides, HDL, and LDL were assessed in the collected serum samples using commercial kits. The kits were purchased from Biomaghreb (Tunisia) and used according to the instructions of the company.

### Thiobarbituric Acid Reactive Substances (TBARS) Measurements

Thiobarbituric acid reactive substances (TBARS), as an indicator of lipid peroxidation, were studied in the liver and kidney tissues as described by Buege and Aust (1978). Overall, a mixture composed of 200 mL of tissue extracts, 250 mL of TCA-BHT (20%TCA and BHT 1%) and 150 mL of TBS (Tris 50 mM, NaCl 150 mM, pH 7.4) was vortexed and then centrifuged for 10 min at 1,500 rpm. To 400 mL of the supernatant, 320 mL of Tris–TBA (Tris 26 mM and TBA 120 mM) and HCl 0.6 N were added. Later on, the mixture was heated for 10 min at 80°C then the absorbance was read at 530 nm. The extinction coefficient of 1.56 × 10^5^ mM^–1^ cm^–1^ was used to determine the level of TBARS.

### Antioxidant Enzymes Studies

Colorimetric methods were used to determine the levels of different enzymatic antioxidants. SOD activity was assessed using the oxidizing reaction of nitroblue tetrazolium (NBT) as previously reported by Beyer and Fridovich (1987). CAT activity was measured using H_2_O_2_ as reported by Aebi (1984). GPx activity was measured using H_2_O_2_ as substrate and reduced GSH as published by Flohe and Günzler (1984).

### Histopathological Studies

Liver and kidney tissues were fixed in bouin solution and possessed for standard histological procedure followed by hematoxylin-eosin (H&E) staining of 4–5 μm thick sections. The slides were examined using optic microscope and several histopathological alterations were recorded. The histopathological features include: necrosis, vacuole formation, epithelial degeneration, focal necrosis of renal tubules and reduction of glomerular space in the kidneys. It includes sinusoidal dilatation, congestion of central vein, ballooning and degeneration of hepatocytes in the liver. The following scores were given: (–) for normal appearance (+, ++, and +++) for mild, moderate, and severe changes.

### Statistical Analysis

Statistical analyses were performed using SPSS (version 17). The analyses include one-way analysis of variance (ANOVA) and Tukey’s *post-hoc* test. All data are expressed as mean ± SEM. Differences with *p* < 0.05 were considered statistically significant.

## Results

### Effects on Food Intake and Body Weight

When compared to controls, both food intake and body weight gain were significantly lower in CPF-treated rats (Table 1). However, the adverse effects of CPF were significantly reduced using ACEO or vitamin C.

**TABLE 1:** Changes in food intake and body weight gain of control and treated rats with chlorpyrifos, *Artemisia campestris* essential oil (ACEO), vitamin C, and their combination (ACEO + CPF and VitC + CPF).

Parameters and treatments	Food intake (g/rat/d)	Gain weights (%)
Control	18.39 ± 1.33	35.33 ± 1.32
ACEO	19.25 ± 1.67	37.22 ± 2.24
CPF	13.59 ± 1.39**	19.53 ± 1.64**
ACEO + CPF	17.89 ± 3.22^##^	34.76 ± 2.66^##^
VitC	18.65 ± 1.28	36.43 ± 1.62
VitC + CPF	18.14 ± 2.29^##^	34.22 ± 1.39^##^

Values are mean ± SEM; n = 6 rats in each group. Statistical significant variations are compared as follows: CPF, ACEO, ACEO + CPF, VitC, and VitC + CPF treated groups compared to control group; ** indicates highly significantly different when p < 0.01, CPF group compared to (ACEO + CPF and VitC + CPF) group: ^##^ indicates highly significantly different when p < 0.01.

### Serum Biochemical Parameters

Levels of AST, ALT, ALP, and LDH in the different group of rats are given in Table 2. Statistical increases of serum AST, ALT, ALP, and LDH in rats exposed to CPF (8.42, 17.46, 22.9, and 25.19%, respectively) compared with control rats. Significant decreases in the levels of serum AST, ALT, ALP and LDH in ACEO plus CPF (ACEO + CPF) rats (3.59, 9.42, 24.9, and 19.76%, respectively) compared with CPF rats. The levels of serum AST, ALT, ALP, and LDH were statistically unchanged in rats treated with ACEO and vitamin C as compared with controls. Levels of serum creatinine, urea, uric acid, cholesterol, triglycerides, HDL, LDL and glucose in control and treated rats are shown in Table 3. Levels of serum creatinine, urea, uric acid, cholesterol, triglycerides, LDL and glucose were statistically enhanced (*p* < 0.05) in rats exposed to CPF and rats exposed to ACEO plus CPF (ACEO + CPF) as compared with control rats. However, level of serum HDL was statistically decreased (*p* < 0.05) in rats exposed to CPF and rats exposed to ACEO associated CPF (ACEO + CPF) as compared with control rats. Significant decreases in the levels of serum creatinine, urea, uric acid, cholesterol, triglycerides, LDL, and glucose in ACEO plus CPF (ACEO + CPF) rats (*p* < 0.05) as compared with CPF rats. In comparison with control data, the levels of serum creatinine, urea, uric acid, cholesterol, triglycerides, HDL, LDL, and glucose were statistically unchanged in rats treated with ACEO and vitamin C.

**TABLE 2:** Changes in the activities of serum enzyme of control and treated rats with chlorpyrifos, *Artemisia campestris* essential oil (ACEO), vitamin C, and their combination (ACEO + CPF and VitC + CPF).

Enzyme (U/L)	Experimental groups					
	Control	ACEO	CPF	ACEO + CPF	VitC	VitC + CPF
AST	261.5 ± 49.32	267.5 ± 8.38	285 ± 15.14*	274.75 ± 27.73^#^	259 ± 31.4	262.66 ± 14.67^#^
ALT	52 ± 5.03	55 ± 2.77	63.66 ± 0.66*	57.66 ± 4.8^#^	56.5 ± 7.5	61.66 ± 2.18
ALP	212.4 ± 45.23	208.33 ± 6	275 ± 14**	206.5 ± 7.5^##^	213.5 ± 8.5	217 ± 43^#^
LDH	1847.5 ± 228.69	1802 ± 83.84	2469 ± 104**	1981 ± 152.97^#^	1779 ± 86	2011.33 ± 102.04^#^

The biochemical parameters: AST (aspartate aminotransferase), ALT (alanine aminotransferase), ALP (alkaline phosphatase), and LDH (lactate dehydrogenase) represented in international units per liter (U/L). Values are mean ± SEM; n = 6 rats in each group. Statistical significant variations are compared as follows: CPF, ACEO, ACEO + CPF, VitC, and VitC + CPF treated groups compared to control group; * indicates significantly different when p < 0.05, ** indicates highly significantly different when p < 0.01, CPF group compared to (ACEO + CPF and VitC + CPF) group; ^#^ indicates significantly different when p < 0.05, ^##^ indicates highly significantly different when p < 0.01.

**TABLE 3:** Means of plasma biochemistry of control and treated rats with chlorpyrifos, *Artemisia campestris* essential oil (ACEO), vitamin C, and their combination (ACEO + CPF and VitC + CPF).

Parameter	Experimental groups					
	Control	ACEO	CPF	ACEO + CPF	VitC	VitC + CPF
Creatinine (μmol/L)	27.3 ± 2.85	29.78 ± 0.57	36.45 ± 1.21**	30.47 ± 0.025^#^	33.15 ± 0.29	32.09 ± 1.84
Urea (mmol/L)	7.3 ± 0.4	7.73 ± 0.57	8.4 ± 0.5*	7.9 ± 0.1^#^	7.8 ± 0.5	8.2 ± 0.4
Uric acid (μmol/L)	81.91 ± 3.23	77.06 ± 4.18	91.39 ± 3.08*	82.06 ± 1.05^#^	81.04 ± 3.45	84.87 ± 2.08^#^
Cholesterol (mmol/L)	1.36 ± 0.18	1.33 ± 0.27	1.5 ± 0.07*	1.38 ± 0.18^#^	1.31 ± 0.15	1.35 ± 0.07^#^
Triglycerides (mmol/L)	1.15 ± 0.26	1.25 ± 0.11	1.8 ± 0.11*	1.31 ± 0.25^#^	1.23 ± 0.04	1.28 ± 0.04^#^
HDL (mmol/L)	0.44 ± 0.03	0.43 ± 0.01	0.37 ± 0.01*	0.41 ± 0.01^#^	0.41 ± 0.08	0.44 ± 0.02^#^
LDL (mmol/L)	0.53 ± 0.01	0.5 ± 0.14	0.64 ± 0.14*	0.52 ± 0.005^#^	0.48 ± 0.1	0.5 ± 0.03^#^
Glucose (mmol/L)	7.3 ± 1.02	7.62 ± 0.25	8.5 ± 1.6*	7.25 ± 0.45^#^	7.9 ± 1.43	6.57 ± 0.17^#^

Values are mean ± SEM; n = 6 rats in each group. Statistical significant variations are compared as follows: CPF, ACEO, ACEO + CPF, VitC, and VitC + CPF treated groups compared to control group; * indicates significantly different when *p* < 0.05, ** indicates highly significantly different when *p* < 0.01, CPF group compared to (ACEO + CPF and VitC + CPF) group; ^#^ indicates significantly different when p < 0.05.

### Oxidative Damage Analyses

The Table 4 exhibits data concerning liver and kidney oxidative stress analyses (SOD, CAT, and GPx activities) and lipid peroxidation. Significant reductions (*p* < 0.05) in hepatic and kidney activities of SOD, CAT, and GPx associated an increase of lipid peroxidation rate have been outlined in CPF group as compared to control. The treatment of rats with ACEO plus CPF showed significant increases (*p* < 0.05) in rat hepatic and kidney antioxidant enzyme activities (SOD, CAT, and GPx) and a decrease in lipid peroxidation rate as compared with CPF rats. Once compared with control rats, no significant alterations in either antioxidant enzyme activities (SOD, CAT, and GPx) or lipid peroxidation rate were observed in rats treated with ACEO, ACEO plus CPF, vitamin C, or vitamin C plus CPF.

**TABLE 4:** Effects of chlorpyrifos, *Artemisia campestris* essential oil (ACEO), vitamin C, and their combination (ACEO + CPF and VitC + CPF) on TBARS levels and the activities of enzymatic antioxidants in liver and kidney of control (C) and experimental rats.

Treatment and parameters		Control	ACEO	CPF	ACEO + CPF	VitC	VitC + CPF
TBARS (nmol MDA/mg protein)	Liver	0.61 ± 0.06	0.61 ± 0.09	1.12 ± 0.1**	0.58 ± 0.06^#^	0.58 ± 0.09	0.57 ± 0.09^#^
	Kidney	2.88 ± 0.11	3.44 ± 0.66	5.04 ± 1.14**	3.55 ± 0.17^##^	3.39 ± 0.31	3.67 ± 0.42^##^
SOD (Units/mg protein	Liver	19.73 ± 1.21	14.31 ± 3.95	11.8 ± 1.14**	17.56 ± 2.29^##^	13.99 ± 1.75	15.44 ± 1.42^##^
	Kidney	21.48 ± 4.28	23.95 ± 3.3	18.03 ± 3.31*	20.32 ± 3.2^#^	25.98 ± 3.15	16.43 ± 2.48^#^
CAT (μmol H_2_O_2_/min/mg protein)	Liver	198.8 ± 10.03	177.59 ± 6.33	142.97 ± 7.34**	185.37 ± 8.8^##^	183.6 ± 4.7	197.61 ± 5.22^##^
	Kidney	100.62 ± 11.04	86.31 ± 8.6	74.87 ± 6.34**	97.23 ± 6.61^##^	98.25 ± 9.46	92.17 ± 7.56^##^
GPx (μmol GSH/min/mg protein)	Liver	2.32 ± 0.27	2.53 ± 0.23	1.4 ± 0.48*	2.08 ± 0.48^##^	2.48 ± 0.3	2.06 ± 0.13^##^
	Kidney	8.35 ± 0.72	7.75 ± 0.37	4.32 ± 0.4**	6.61 ± 1.04^##^	7.90 ± 1.31	6.79 ± 1.2

Values are mean ± SEM; n = 6 rats in each group. Statistical significant variations are compared as follows: CPF, ACEO, ACEO + CPF, VitC, and VitC + CPF treated groups compared to control group; * indicates significantly different when p < 0.05, ** indicates highly significantly different when p < 0.01, CPF group compared to (ACEO + CPF and VitC + CPF) group; ^#^ indicates significantly different when p < 0.05, ^##^ indicates highly significantly different when p < 0.01.

### Histopathological Examinations

**FIGURE 2:**
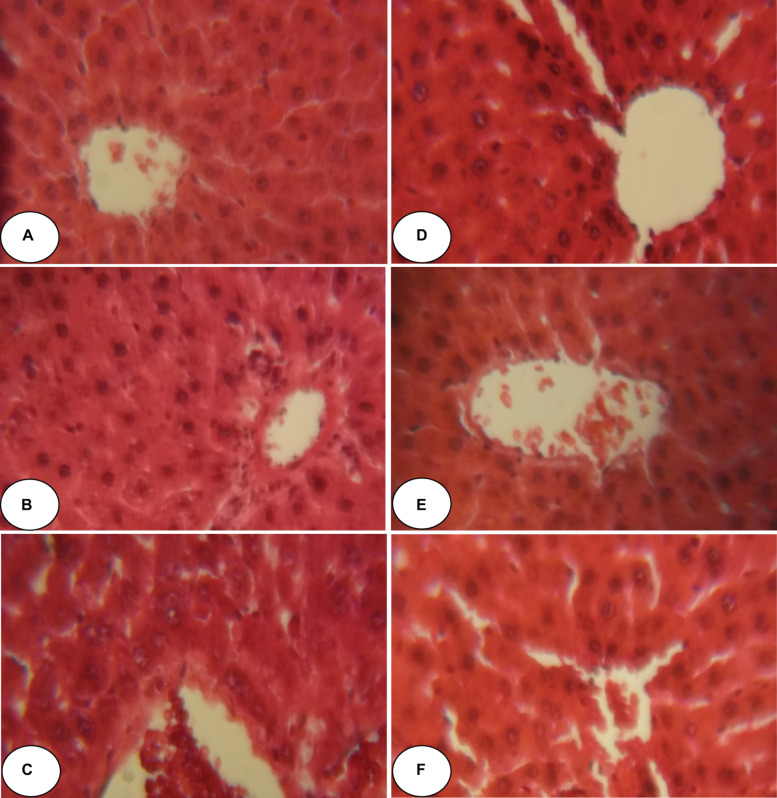
Histopathological observation of rat hepatic tissue of control, ACEO, chlorpyrifos, and vitamin C stained with hematoxylin and eosin viewed at original magnification (×400). **(A)** Control liver showing normal architecture; **(B)** ACEO alone administered rats showing normal architecture in liver tissue; **(C)** CPF liver sections showed abnormal cellular morphology accompanied by sinusoidal dilatation, congestion, ballooning, necrosis, vacuole formations, and degeneration of hepatocytes after chlorpyrifos treatment; **(D)** ACEO + CPF co-treated rats indicating protected hepatic tissue; **(E)** VitC alone treated rats showed normal architecture in liver tissue; and **(F)** VitC + CPF showed that vitamin C co-administration restored liver tissue alteration induced by chlorpyrifos.

**FIGURE 3:**
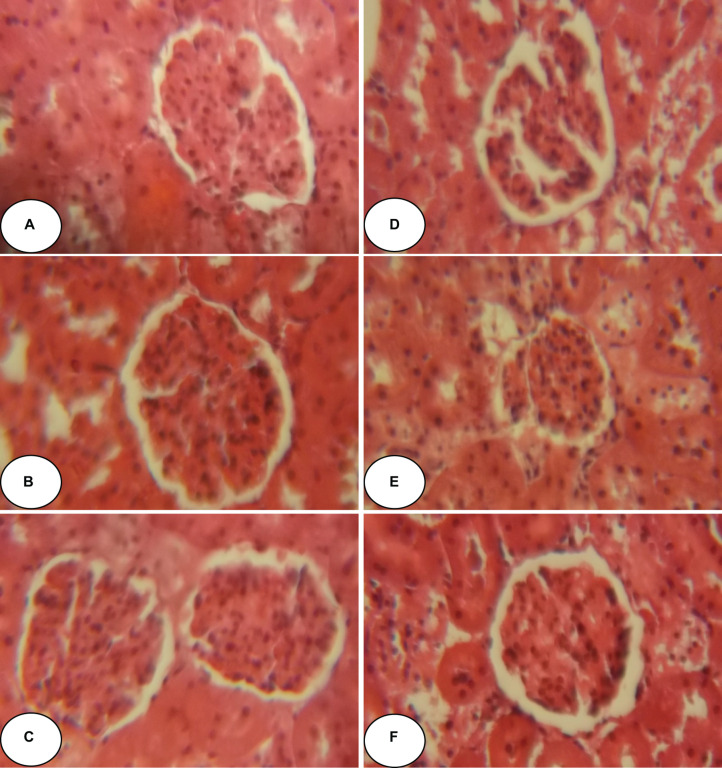
Histopathological observation of renal tissue of control, ACEO, chlorpyrifos, and vitamin C stained with hematoxylin and eosin viewed at original magnification (×400). **(A)** Control kidney showing normal architecture; **(B)** ACEO alone administered rats showing normal architecture in renal tissue; **(C)** CPF kidney sections revealed tubular epithelial degenerations, focal necrosis of renal tubules, vacuole formations and reduction of glomerular space after chlorpyrifos treatment; **(D)** ACEO + CPF showed that ACEO co-administration restored renal tissue alteration induced by chlorpyrifos; **(E)** VitC alone treated rats showed normal architecture in kidney tissue; and **(F)** VitC + CPF showed that vitamin C co-administration restored renal tissue damage induced by chlorpyrifos.

Figures 2, 3 and Table 5 show histopathological findings and attributed scores for both liver and kidneys in all the experimental rats. Liver tissue sections revealed sinusoidal dilatation, congestion, ballooning, necrosis, vacuole formations, and degeneration of hepatocytes after chlorpyrifos treatment. Both ACEO and vitamin C restored back the liver histological alterations, which were induced by CPF. However, the control and the rats treated with ACEO or vitamin C showed normal architecture of the hepatic lobule (Figure 2). Renal tissue sections revealed tubular epithelial degenerations, focal necrosis of renal tubules, vacuole formations and reduction of glomerular space after chlorpyrifos treatment. Here again, ACEO and vitamin C co-administration alleviated renal histological alterations which were induced by CPF. However, the control rats and the treated rats with ACEO and vitamin C showed normal architecture of the renal parenchyma (Figure 3).

**TABLE 5:** The severity of the reaction in liver and kidney tissue of control and treated rats with chlorpyrifos, *Artemisia campestris* essential oil (ACEO), vitamin C, and their combination (ACEO + CPF and VitC + CPF).

Histopathological alterations	Experimental groups					
	Control	ACEO	CPF	ACEO + CPF	VitC	VitC + CPF
**Liver**						
Sinusoidal dilatation	–	–	++	–	–	–
Congestion of central vein	–	–	+	+	–	+
Ballooning degeneration of hepatocytes	–	–	++	+	–	–
Necrosis	–	–	+	–	–	–
Vacuole formations	–	–	++	–	–	+
**Kidneys**						
Epithelial degenerations	–	–	++	+	–	–
Focal necrosis of renal tubules	–	–	++	–	–	+
Vacuole formations	–	–	+	+	–	+
Reduction of glomerular space	–	–	+	–	–	–

*+++ Severe; ++ moderate; + mild; – nil.*

## Discussion

In the current study, rats exposed to CPF exhibited increases of serum AST, ALT, ALP, and LDH activities, which could be a consequence of hepatic tissues injury. The increase of these liver biomarkers within the serum is usually indicating damage in both liver morphology and function (Paradies et al., 2014; Rahmouni et al., 2019; Badraoui et al., 2020). Moreover, the elevation of serum transaminases (AST and ALT) following CPF treatment disturbed not only the synthesis of energetic macromolecules, which are needed for several vital and metabolic functions but also the detoxification processes (Ali et al., 2016). Our results are in agreement of the studies of Uzun and Kalender (2013), which demonstrated that CPF induced hepatotoxic and hematologic changes in rats. So far, these enzymatic increases could be associated with liver dysfunction as well as severe alteration in the permeability of the liver membrane.

Serum creatinine, uric acid, and urea indicators of kidney function increased significantly in rats after CPF treatment which may indicate degradation of both purines and pyrimidines and impaired kidney function. Many reports investigated an increase of creatinine, urea and uric acid in serum of rats exposed to toxic compounds including pesticides (Badraoui et al., 2007, 2012; Hakim et al., 2008; Amri et al., 2017; Meligi and Hassan, 2017; Kasteel et al., 2020). Levels of serum cholesterol, triglycerides, LDL, and glucose were statistically enhanced in rats exposed to CPF. Our results corroborates with the studies of El-Demerdash and Nasr (2014), which demonstrated that diazinon disrupted several biochemical parameters including lipid profile. In fact, it is believed that lipids (particularly the unsaturated fatty acids which belong to the main components of the cell membranes) belong to the most sensitive molecules to oxidizing stress. Many reports showed significant changes in lipids profile in both blood and tissues of experimental animals exposed to pests (García-García et al., 2016; Rahmouni et al., 2019; Badraoui et al., 2020). On the other hand, this enhancement of serum cholesterol in rats treated with CPF may be due to liver damage that can be attributed to the effect of CPF on the permeability of liver cell membrane. Our study showed that all the serum hepatic, and renal lipid profile markers, and the blood glucose were restored to normal levels in the CPF + ACEO and CPF + VitC groups. This may be due to the antioxidant activities (Saoudi et al., 2017b) of the bioactive components of ACEO. Recent reports were performed on the antioxidant activities of essential oil of *A. campestris* and its effects on many biological studies (antioxidant, vasorelaxant, antiplatelet, anticancer, anti-acetylcholinesterase,…). In the current study, the perturbations of the biochemical parameters (hepatic markers, kidney markers, lipid profile, and blood glucose) were associated with an oxidative damage in rats’ livers and kidneys after treatment with CPF. Our results revealed that CPF treatment caused oxidative stress in the liver and kidney of rats, which was associated with the generation of lipid peroxidation (LPO). The overproduction of LPO disturbed the integrity of cellular membranes and implicated in the pathogenesis of liver and kidney injuries (Badraoui et al., 2007; Joshi et al., 2017; Saoudi et al., 2018). For this reason, it has been used as an indicator of pesticides induced oxidative stress (Badraoui et al., 2007; Hemalatha et al., 2016; Mzid et al., 2017). The present results demonstrated that the administration of CPF caused statistically increases in the level of liver and kidney LPO and decreases in antioxidant activities such as SOD, CAT, and GPx. These findings are consistent with previous studies, which indicated that CPF and other pests caused oxidative damage confirmed by a decrease in the levels of antioxidant enzymes (SOD, CAT, and GPx), and increase in the level of LPO (Mansour and Gamet-Payrastre, 2016; Amri et al., 2017; Saoudi et al., 2017a). The increase of LPO and decreases in SOD, CAT, and GPx activities observed in hepatic and kidneys tissues following CPF exposure could probably be explained by an excessive production of ROS, which could be related to hepatocyte and kidney enzymes leakage (Narra et al., 2017). The present results are in accordance with those of Mosbah et al. (2016) who demonstrated a significant increase in LPO level and decreases of SOD, CAT, and GPx activities in plasma and testis after treatment of male rats with CPF. In fact, CPF can be responsible for increasing the production of reactive oxygen species (ROS) in cells, which lead to increased lipid peroxidation levels and oxidative damage (Kasteel et al., 2020). The decrease in antioxidant enzyme activities in liver and kidney tissues was due to cellular injury and death of healthy cells that are able to respond to the oxidative insult. On the other hand, it may be due to the insufficient detoxification capacity of CPF and damage caused by ROS (Amri et al., 2017; Kutluyer et al., 2017; Mzid et al., 2017). SOD, CAT, and GPx activities, as antioxidant enzymes, play an important role in the protection against the deleterious effects of LPO by scavenging superoxide anions and hydroxyl ions. ACEO was also reported to combat effects of many toxins, including pesticides and heavy metals (Badraoui et al., 2012; Houicher et al., 2016; Saoudi et al., 2017a). In our experiment, pre-administration of ACEO or vitamin C against CPF resulted in an increase in the activities of SOD, catalase and GPx in the liver and kidney tissues of rats. Preventive treatment with ACEO also restored the MDA level suggesting decrease in lipid peroxidation. These properties could be attributed to the high levels of antioxidants such as phyocyanin, carotenoids, vitamins, minerals, lipids, proteins and carbohydrates, which have been reported in *A. campestris* (Dib et al., 2017). Therefore, ACEO could be used to prevent hepatic and renal diseases especially those induced by oxidative damage (Saoudi et al., 2017a). The changes in oxidative stress, liver, and kidney biomarkers in rats exposed to the CPF corroborated the histopathological lesions observed in this study. In fact, the liver histological observations revealed sinusoidal dilatation, congestion, ballooning, necrosis, vacuole formations, and degeneration of hepatocytes after CPF treatment. The present findings are in consequence with the results of previous investigations after exposure to different types of insecticides (Badraoui et al., 2007; Singh et al., 2017; Rahmouni et al., 2019). The kidney histological examinations revealed tubular epithelial degenerations, focal necrosis of renal tubules, vacuole formations, and reduction of glomerular space after CPF treatment. Histological observations of the kidneys of CPF treated rats were comparable with other studies conducted on deltamethrin treated rats (Saoudi et al., 2017a). The observations in the previous mentioned studies are in corroboration and support our results. The marked changes in the overall histoarchitecture of the liver and kidney in response to CPF could be attributed to the toxic effects of the insecticide through the excessive generation of ROS, causing damage to various membrane components of the hepatic and renal tissues.

ACEO co-administration ameliorates the histological alterations induced by CPF, suggesting an efficient antiradical/antioxidant of ACEO. Moreover, our results are in accordance with earlier studies that postulated the beneficial role of ACEO on the histopathological and biochemical changes in rats (Saoudi et al., 2017a). We can conclude that CPF induced liver and kidney oxidative damage in male rats. The administration of essential oil of *A. campestris* provided significant protection against chlorpyrifos-induced oxidative stress, biochemical disruptions and histopathological alterations.

## Data Availability Statement

All datasets generated for this study are included in the article/supplementary material, further inquiries can be directed to the corresponding author/s.

## Ethics Statement

All animal experiments were conducted according to the Ethical Committee Guidelines for the care and use of laboratory animals of our institution (Faculty of Sciences of Sfax, Tunisia).

## Author Contributions

MS, FR, RB, and AE: *in vivo* investigation, data collection and analysis, supervision, conceptualization, writing—original draft, writing—review and editing, and visualization. KJ: biochemical analysis. All authors contributed to the article and approved the submitted version.

## Conflict of Interest

The authors declare that the research was conducted in the absence of any commercial or financial relationships that could be construed as a potential conflict of interest.

## References

[B1] Abolaji A. O. Awogbindin I. O. Adedara I. A. Farombi E. O. (2017a). Insecticide chlorpyrifos and fungicide carbendazim, common food contaminants mixture, induce hepatic, renal, and splenic oxidative damage in female rats. *Hum. Exp. Toxicol.* 36 483–493. 10.1177/0960327116652459 27268782

[B2] Abolaji A. O. Ojo M. Afolabi T. T. Arowoogun M. D. Nwawolor D. Farombi E. O. (2017b). Protective properties of 6-gingerol-rich fraction from *Zingiber officinale* (Ginger) on chlorpyrifos-induced oxidative damage and inflammation in the brain, ovary and uterus of rats. *Chem. Biol. Interact.* 270 15–23. 10.1016/j.cbi.2017.03.017 28373059

[B3] Aebi H. (1984). Catalase in vitro. *Methods Enzymol.* 105 121–126. 10.1016/s0076-6879(84)05016-3 6727660

[B4] Akacha A. Badraoui R. Rebai T. Zourgui L. (2020). Effect of *Opuntia ficus indica* extract on methotrexate-inducedtesticular injury: a biochemical, docking and histological study. *J. Biomol. Struct. Dyn.* 11 1–11. 10.1080/07391102.2020.1856187 33305699

[B5] Akrout A. Gonzalez L. A. El Jani H. Madrid P. C. (2011). Antioxidant and antitumor activities of *Artemisia campestris* and *Thymelaea hirsuta* from southern Tunisia. *Food Chem. Toxicol.* 49 342–347. 10.1016/j.fct.2010.11.003 21075159

[B6] Albuquerquea E. Burkea R. Rao Gullapallib J. Mamczarza E. P. (2017). Developmental neurotoxicity of the organophosphorus pesticide chloryprifos: from animal behavior to molecular mechanisms. *J. Neurochem.* 142 188–225. 10.1111/jnc.14077 PMC5673499 28791702

[B7] Ali S. A. Mohamed A. A. R. Ali H. Elbohi K. M. (2016). Sublethal effect of fipronil exposure on liver and kidney tissues with evaluation of the recovery ability of Japanese quail (*Coturnix japonica*). *Jpn. J. Vet. Res.* 64 S131–S138.

[B8] Amri N. Rahmouni F. Chokri M. A. Rebai T. Badraoui R. (2017). Histological and biochemical biomarkers analysis reveal strong toxicological impacts of pollution in hybrid sparrow (*Passer domesticus* × *Passer hispaniolensis*) in southern Tunisia. *Environ. Sci. Pollut. Res.* 24 17845–11785. 10.1007/s11356-017-9352-3 28612310

[B9] Asghari M. H. Moloudizargari M. Bahadar H. Abdollahi M. (2017). A review of the protective effect of melatonin in pesticide-induced toxicity. *Expert Opin. Drug Metab. Toxicol.* 13 545–554. 10.1080/17425255.2016.1214712 27434705

[B10] Badraoui R. Ben-Nasr H. Bardakci F. Rebai T. (2020). Pathophysiological impacts of exposure to an endocrine disruptor (tetradifon) on α-amylase and lipase activities associated metabolic disorders. *Pestic. Biochem. Physiol.* 167:104606. 10.1016/j.pestbp.2020.104606 32527427

[B11] Badraoui R. Blouin S. Moreau M. F. Gallois Y. Rebai T. Sahnoun Z. (2009). Effect of alpha tocopherol acetate in Walker 256/B cells-induced oxidative damage in a rat model of breast cancer skeletal metastases. *Chem. Biol. Interact.* 182 98–105. 10.1016/j.cbi.2009.09.010 19781538

[B12] Badraoui R. Nasr H. B. Louati R. Ellouze F. Rebai T. (2012). Nephrotoxic effect of tetradifon in rats: a biochemical and histomorphometric study. *Exp. Toxicol. Pathol.* 64 645–650. 10.1016/j.etp.2010.12.008 21216578

[B13] Badraoui R. Sahnoun Z. Abdelmoula N. B. Hakim A. Fki M. Rebai T. (2007). May antioxidant status depletion by tetradifon induce secondary genotoxicity in female wistar rats via oxidative stress? *Pestic. Biochem. Physiol.* 88 149–155. 10.1016/j.pestbp.2006.10.007

[B14] Bebe F. N. Panemangalore M. (2003). Exposure to low doses of endosulfan and chlorpyrifosmodifie endogenous antioxidants in tissues of rats. *J. Environ. Sci. Health B* 38 349–363. 10.1081/pfc-120019901 12716052

[B15] Beyer W. F. Jr. Fridovich I. (1987). Assaying for superoxide dismutase activity: some large consequences of minor changes in conditions. *Anal. Biochem.* 161 559–566. 10.1016/0003-2697(87)90489-1 3034103

[B16] Burke R. D. (2016). *A Structural, Functional, and Behavioral Evaluation of the Developmental Neurotoxicity of the Organophosphorus Insecticide Chlorpyrifos in Guinea Pigs: Mechanistic Implications.* Doctoral dissertation, University of Maryland, Baltimore, MD.

[B17] Buege J. A. Aust S. D. (1978). Microsomal lipid peroxidation. *Methods Enzymol.* 52 302–310. 672633 10.1016/s0076-6879(78)52032-6

[B18] de Bon H. Huat J. Parrot L. Sinzogan A. Martin T. Malézieux E. (2014). Pesticide risks from fruit and vegetable pest management by small farmers in sub-Saharan Africa. a review. *Agron. Sustain. Dev.* 34 723–736. 10.1007/s13593-014-0216-7

[B19] Dib I. Fauconnier M. L. Sindic M. Belmekki F. Assaidi A. Berrabah M. (2017). Chemical composition, vasorelaxant, antioxidant and antiplatelet effects of essential oil of *Artemisia campestris* L. from oriental Morocco. *BMC Complement. Altern. Med.* 17:82. 10.1186/s12906-017-1598-2 28143473 PMC5282690

[B20] Dominah G. A. McMinimy R. A. Kallon S. Kwakye G. F. (2017). Acute exposure to chlorpyrifos caused NADPH oxidase mediated oxidative stress and neurotoxicity in a striatal cell model of Huntington’s disease. *Neurotoxicology* 60 54–69. 10.1016/j.neuro.2017.03.004 28300621

[B21] Donkor A. Osei-Fosu P. Dubey B. Kingsford-Adaboh R. Ziwu C. Asante I. (2016). Pesticide residues in fruits and vegetables in Ghana: a review. *Environ. Sci. Pollut. Res.* 23 18966–18987. 10.1007/s11356-016-7317-6 27530198

[B22] El-Demerdash F. M. Nasr H. M. (2014). Antioxidant effect of selenium on lipid peroxidation, hyperlipidemia and biochemical parameters in rats exposed to diazinon. *J. Trace Elem. Med. Biol.* 28 89–93. 10.1016/j.jtemb.2013.10.001 24188896

[B23] Flohe L. Günzler W. A. (1984). Analysis of glutathione peroxidase. *Methods Enzymol.* 105 114–121. 6727659 10.1016/s0076-6879(84)05015-1

[B24] García-García C. R. Parrón T. Requena M. Alarcón R. Tsatsakis A. M. Hernández A. F. (2016). Occupational pesticide exposure and adverse health effects at the clinical, hematological and biochemical level. *Life Sci.* 145 274–283. 10.1016/j.lfs.2015.10.013 26475762

[B25] Hakim A. Kallel H. Sahnoun Z. Badraoui R. Jammoussi K. Bouaziz M. (2008). Lack of nephrotoxicity following 15-day therapy with high doses of colistin in rats. *Med. Sci. Monit.* 14 BR74–BR77. 18376342

[B26] Hemalatha D. Amala A. Rangasamy B. Nataraj B. Ramesh M. (2016). Sublethal toxicity of quinalphos on oxidative stress and antioxidant responses in a freshwater fish *Cyprinus carpio*. *Environ. Toxicol.* 31 1399–1406. 10.1002/tox.22145 25899319

[B27] Houicher A. Hechachna H. Özogul F. (2016). In vitro determination of the antifungal activity of *Artemisia campestris* essential oil from Algeria. *Int. J. Food Prop.* 19 1749–1756. 10.1080/10942912.2015.1107734

[B28] Joshi D. Mittal D. K. Shukla S. Srivastav S. K. Dixit V. A. (2017). *Curcuma longa* Linn. extract and curcumin protect CYP 2E1 enzymatic activity against mercuric chloride-induced hepatotoxicity and oxidative stress: a protective approach. *Exp. Toxicol. Pathol.* 69 373–382. 10.1016/j.etp.2017.02.009 28336172

[B29] Kasteel E. E. J. Nijmeijer S. M. Darney K. Lautz L. S. Dorne J. L. C. M. Kramer N. I. (2020). Acetylcholinesterase inhibition in electric eel and human donor blood: an in vitro approach to investigate interspecies differences and human variability in toxicodynamics. *Arch. Toxicol.* 94 4055–4065. 10.1007/s00204-020-02927-8 33037899 PMC7655571

[B30] Kutluyer F. Kocabas̨ M. Eris̨ir M. Benzer F. (2017). Effect of the organophosphate insecticide chlorpyrifos exposure on oxidative stress and quality of *Salmo coruhensis* spermatozoa. *Toxin Rev.* 38 1–6.

[B31] Mansour S. A. K. Gamet-Payrastre L. (2016). Ameliorative effect of vitamin E to mouse dams and their pups following exposure of mothers to chlorpyrifos during gestation and lactation periods. *Toxicol. Ind. Health* 32 1179–1196. 10.1177/0748233714548207 25234640

[B32] Meligi N. M. Hassan H. F. (2017). Protective effects of *Eruca sativa* (rocket) on abamectin insecticide toxicity in male albino rats. *Environ. Sci. Pollut. Res.* 24 9702–9712. 10.1007/s11356-017-8671-8 28251533

[B33] Mosbah R. Yousef M. I. Maranghi F. Mantovani A. (2016). Protective role of *Nigella sativa* oil against reproductive toxicity, hormonal alterations, and oxidative damage induced by chlorpyrifos in male rats. *Toxicol. Ind. Health* 32 1266–1277. 10.1177/0748233714554675 25425536

[B34] Mzid M. Badraoui R. Khedir S. B. Sahnoun Z. Rebai T. (2017). Protective effect of ethanolic extract of *Urtica urens* L. against the toxicity of imidacloprid on bone remodeling in rats and antioxidant activities. *Biomed. Pharmacother.* 91 1022–1041. 10.1016/j.biopha.2017.05.023 28531918

[B35] Narra M. R. Rajender K. Reddy R. R. Murty U. S. Begum G. (2017). Insecticides induced stress response and recuperation in fish: biomarkers in blood and tissues related to oxidative damage. *Chemosphere* 168 350–357. 10.1016/j.chemosphere.2016.10.066 27810534

[B36] Negatu B. Kromhout H. Mekonnen Y. Vermeulen R. (2016). Use of chemical pesticides in ethiopia: a cross-sectional comparative study on knowledge, attitude and practice of farmers and farm workers in three farming systems. *Ann. Occup. Hyg.* 60 551–566. 10.1093/annhyg/mew004 26847604

[B37] Paradies G. Paradies V. Ruggiero F. M. Petrosillo G. (2014). Oxidative stress, cardiolipin and mitochondrial dysfunction in nonalcoholic fatty liver disease. *World J. Gastroenterol.* 20 14205–14218. 10.3748/wjg.v20.i39.14205 25339807 PMC4202349

[B38] Petrova S. Mikhailova A. Donchev N. (1992). The effect of elevated amounts of ascorbic acid on the status of the vitamin and lung disorders in guinea pigs inhaling styrene. *Probleminakhigienata* 17 137–145. 1364534

[B39] Radulović N. S. Randjelović P. J. Stojanović N. M. Blagojević P. D. Stojanović-Radić Z. Z. Ilić I. R. (2013). Toxic essential oils. Part II: chemical, toxicological, pharmacological and microbiological profiles of *Artemisia annua* L. volatiles. *Food Chem. Toxicol.* 58 37–49. 10.1016/j.fct.2013.04.016 23607933

[B40] Rahmouni F. Badraoui R. Amri N. Elleuch A. El-Feki A. Rebai T. (2019). Hepatotoxicity and nephrotoxicity in rats induced by carbon tetrachloride and the protective effects of *Teucrium polium* and vitamin. *Toxicol. Mech. Meth.* 29 313–332. 10.1080/15376516.2018.1519864 30676168

[B41] Saoudi M. Badraoui R. Bouhajja H. Ncir M. Rahmouni F. Grati M. (2017a). Deltamethrin induced oxidative stress in kidney and brain of rats: protective effect of *Artemisia campestris* essential oil. *Biomed. Pharm.* 94 955–963. 10.1016/j.biopha.2017.08.030 28810533

[B42] Saoudi M. Hmida I. B. Kammoun W. Rebah F. B. Jamoussi K. Feki A. E. (2018). Protective effects of oil of *Sardinella pilchardis* against subacute chlorpyrifos-induced oxidative stress in female rats. *Arch. Environ. Occup. Health* 73 128–135. 10.1080/19338244.2017.1317627 28394715

[B43] Saoudi M. Ncir M. Ben M. A. Grati M. Jamoussi K. Allouche N. (2017b). Chemical components, antioxidant potential and hepatoprotective effects of *Artemisia campestris* essential oil against deltamethrin-induced genotoxicity and oxidative damage in rats. *Gen. Physiol. Biophys.* 36 331–342. 10.4149/gpb_2016057 28635610

[B44] Sears M. Walker C. R. Van der Jagt R. H. Claman P. (2006). Pesticide assessment: protecting public health on the home turf. *Paediatr. Child Health* 11 229–234. 19030278 PMC2528613

[B45] Sharma S. Chadha P. (2016). Induction of neurotoxicity by organophosphate pesticide chlorpyrifos and modulating role of cow urine. *Springerplus* 5:1344. 10.1186/s40064-016-3004-9 PMC4987744 27588237

[B46] Singh R. Srivastava A. K. Gangwar N. K. Singh U. Singh R. (2017). Ameliorative effect of vitamin E on cypermethrin induced hepatotoxicity and oxidative stress in male Wistar rats. *J. Anim. Res.* 7 445–450. 10.5958/2277-940x.2017.00066.3

[B47] Tanvir E. M. Afroz R. Chowdhury M. A. Z. Gan S. H. Karim N. Islam M. N. (2016). A model of chlorpyrifos distribution and its biochemical effects on the liver and kidneys of rats. *Hum. Exp. Toxicol.* 35 991–1004. 10.1177/0960327115614384 26519480

[B48] Uzun F. G. Kalender Y. (2013). Chlorpyrifos induced hepatotoxic and hematologic changes in rats: the role of quercetin and catechin. *Food Chem. Toxicol.* 55 549–556. 10.1016/j.fct.2013.01.056 23402859

